# Phytochemical Constituents and Antiproliferative Activities of Essential Oils from Four Varieties of Malaysian *Zingiber officinale* Roscoe against Human Cervical Cancer Cell Line

**DOI:** 10.3390/plants11101280

**Published:** 2022-05-10

**Authors:** Atiqah Zaid, Xue Rou Haw, Huda Hisham Alkatib, Sreenivasan Sasidharan, Philip J. Marriott, Yong Foo Wong

**Affiliations:** 1 Centre for Research on Multidimensional Separation Science, School of Chemical Sciences, Universiti Sains Malaysia, Penang 11800, Malaysia; atiqahzaid@student.usm.my (A.Z.); xuerouhaw306@gmail.com (X.R.H.); 2 Institute for Research in Molecular Medicine (INFORMM), Universiti Sains Malaysia, Penang 11800, Malaysia; huda.alkatib22@gmail.com (H.H.A.); srisasidharan@usm.my (S.S.); 3 Australian Centre for Research on Separation Science, School of Chemistry, Monash University, Wellington Road, Clayton, Melbourne, VIC 3800, Australia; philip.marriott@monash.edu

**Keywords:** *Zingiber officinale* Roscoe, essential oil, GC-MS, PCA, antiproliferative, HeLa

## Abstract

This study evaluates the volatile metabolic constituents and anticancer potential of essential oils distilled from the rhizomes of four Malaysian *Zingiber officinale* Roscoe (Zingiberaceae family) varieties (Bentong (BE), Cameron Highlands (CH), Sabah (SA), and Bara (BA)). The ginger essential oils were analyzed by gas chromatography coupled with quadrupole mass spectrometry (GC qMS). A total of 58 secondary compounds were tentatively identified, representing 82.6–87.4% of the total ion count. These metabolites comprise mainly of monoterpene hydrocarbons (19.7–25.5%), oxygenated monoterpenes (23.6–33.7%), sesquiterpene hydrocarbons (21.3–35.6%), oxygenated sesquiterpenes (1.5–3.9%), and other minor classes of compounds (0.7–2.7%). Principal component analysis (PCA) enabled differentiation of the analyzed ginger essential oils according to their varieties, with respect to their metabolites and relative quantities. The antiproliferative activity against the HeLa cervical cancer cell line was investigated via the 3-(4,5-dimethylthiazol-2-yl)-2,5-diphenyltetrazolium bromide (MTT) assay. The oils were found to exhibit strong antiproliferative activities with IC_50_ values of 23.8, 35.3, 41.3, and 42.5 μg/mL for BA, BE, SA, and CH, respectively. These findings suggest that the differences among the secondary metabolites and their abundance in different varieties of *Z. officinale* essential oils appear to be related to their antiproliferative potential. The strong antiproliferative effects of these oils signified their potential in the prevention and chemotherapy of cervical carcinoma treatment.

## 1. Introduction

*Zingiber officinale* Roscoe is a well-known herb belonging to the Zingiberaceae family which consists of 53 genera and approximately 1300 species [1,2]. This perennial rhizomatous plant only propagates through its rhizome (colloquially referred to as ginger), with physical appearances as pale yellowish, thick-lobed, with tuberous joints [2]. This flowering plant is seasonally cultivated in many tropical and subtropical regions throughout the world, including Australia, China, Indonesia, India, Malaysia, Nigeria, Spain, and others [3,4]. Albeit both ginger flowers and bruised stems have a pleasant aroma, the rhizomes are most often used as a spice and condiment due to its spicy flavor and woody aroma. Apart from being one of the most internationally traded spices, the *Z. officinale* rhizome is also highly valued for its health-promoting properties. Ginger rhizomes have been extensively documented as folk and traditional medicines for the treatment of numerous ailments, such as asthma, dyspepsia, heart palpitation, rheumatism, and vomiting [1,5].

Bioactive molecules that reside within the ginger rhizomes include volatile terpenic compounds, anthocyanin, phenolic compounds, and others. Notably, ginger essential oil has very high commercial and application value due to its dietary value and beneficial effects in therapeutic treatments. Previous phytochemical assessment of ginger essential oil identified a range of monoterpenes, sesquiterpenes, and their oxygenated derivatives, along with some aldehydes and alcohols [1,6,7]. These volatile secondary compounds were reported to exhibit significant pharmacological properties, such as antibacterial, anticancer, antifungal, anti-inflammatory, antioxidant, immunomodulatory, and others [8,9,10]. For these reasons, these oils have attracted much interest of pharmaceutical, cosmetics, and food industries in recent years.

In Malaysia, Bara (BA), Bentong (BE), Cameron Highlands (CH), and Sabah (SA) gingers are the commercially cultivated *Z. officinale* varieties. Among the four ginger varieties, Bentong and Bara gingers are highly valued by locals due to their unique and strong aroma [11]. In particular, Bentong ginger can be characteristically identified by its large rhizome and high pungency, and is locally known as the ‘Emperor of Ginger’ and was recognized as one of the Pahang’s Geographical Indications by the Agriculture Bureau of Pahang in 2015. On the contrary, Bara ginger is rather unique as compared to the other varieties, mostly because of its small, reddish appearance, as well as its superior pharmacological properties, attributable to its high level of vanilloids, phenolics, and flavonoids [12,13,14].

Despite immense strides in modern medicine, the escalation in cancer mortality and limitations of conventional approaches to treat the dreaded disease without any after-effect has increased the interest in natural nutraceuticals. In recent years, the use of *Z. officinale* oil as an alternate anticancer regimen has garnered considerable attention, leading to multiple reports suggesting inhibition of cancer cell lines by inducing apoptosis and constraining proliferation [15,16,17]. The cytotoxicity of the oil has previously been reported against a variety of cancer cell lines, which include L929 (mouse lung fibroblast), Dalton’s Lymphoma Ascites (DLA), Ehrlich Ascites Carcinoma (EAC), Vero cells, human prostate cancer cells (LNCaP and PC-3), human glioblastoma cells SF-(767 and SF-763), and human breast cancer cells (MCF-7, MDA-MB-175, MDA-MB-231, and MDA-MB-468) [18,19,20].

Currently, only limited studies have been conducted to investigate the phytoconstituents and anticancer potentials of certain varieties of *Z. officinale* rhizome essential oils available in Malaysia. Therefore, the present study aimed at: (i) studying the metabolic composition of four varieties of Malaysian ginger rhizome (BE, CH, SA, and BA) essential oils, and (ii) evaluating their antiproliferative potential against the human adenocarcinoma cervical cancer (HeLa) cell line via the MTT assay. To the best of our knowledge, this is the first report that deals with the comparative studies of antiproliferative activities and variation of secondary compounds in BE, CH, SA, and BA ginger essential oils. This study provides valuable information for the continued development of steam-distilled ginger essential oils as potential chemopreventive and chemotherapeutic agents for cervical carcinoma.

## 2. Results and Discussion

### 2.1. Variation in Chemical Composition of BE, CH, SA, and BA Rhizomes Essential Oils

Steam-distillation of four varieties of *Z. officinale* rhizomes (BE, CH, SA, and BA) yielded yellow-colored essential oils, with a variation in content ranging from 0.08% to 0.77% (*w*/*w*) on the basis of fresh weight, obtained in the following order: BA (0.15%) > BE (0.77%) > SA (0.10%) > CH (0.08%). Since the therapeutic values of ginger essential oil are attributable to its accumulated volatile secondary compounds, detailed chemical profiling of BE, CH, SA, and BA ginger essential oils is needed to understand its medicinal characteristics. Thus, GC-MS was utilized for untargeted metabolomic analysis of the volatile secondary compounds present in the extracted essential oils. Out of 78 detected components, 58 were tentatively identified, corresponding to 82.6% to 87.3% of the total ion counts of the analyzed oils (Table S1). Interestingly, only 48 components appeared to be commonly identified across all varieties. The components were classified into three major groups of terpenoids (monoterpenes, sesquiterpenes, and their derivatives) dominating the ginger oil composition, and four minor groups of non-terpenoids (alcohols, ketones, acetates, and hydrocarbons). The characteristic GC-MS total ion chromatograms (TICs) of the volatile profiles of the four *Z. officinale* essential oils are depicted in Figure S1. A total of 17 major compounds (≥1.0%) were tentatively identified in the analyzed ginger rhizome essential oils, including 4 monoterpene hydrocarbons, 11 oxygenated monoterpenes, 5 sesquiterpene hydrocarbons, and 1 oxygenated sesquiterpene (Table 1).

**Figure 1:**
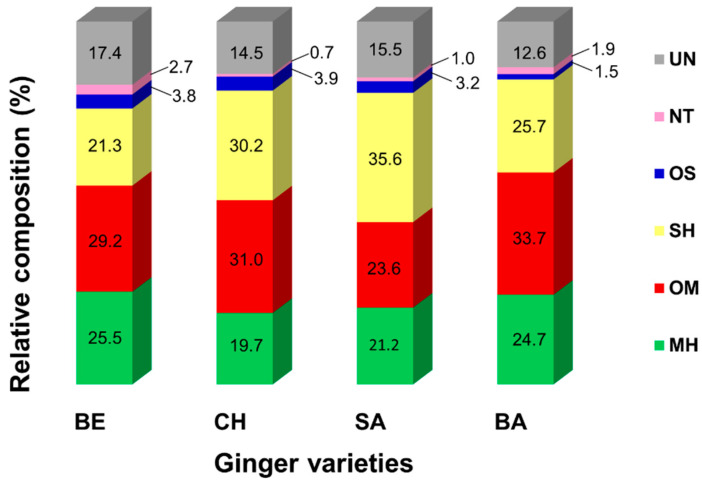
Relative metabolic composition (%) of the analyzed *Z. officinale* essential oils: Bentong (BE), Cameron Highlands (CH), Sabah (SA), and Bara (BA). The constituents consist of: monoterpenic hydrocarbons (MH), oxygenated monoterpenes (OM), sesquiterpenic hydrocarbons (SH), oxygenated sesquiterpenes (OS), non-terpenic compounds (NT), and unidentified compounds (UN).

A summary of the overall metabolic composition of the analyzed *Z. officinale* oils is illustrated in Figure 1. The relative percentage of each component class differed from one variety to another, though a similar compositional pattern of terpenoids could be discerned. Monoterpenoids predominated the oils’ content (44.7–58.4%), with α-citral (7.3–9.8%; or geranial) being a major contributor to the high monoterpenoid level in BE, CH, and SA, while camphene (10.5%) was the major monoterpene in BA. The total content of monoterpenoids in BE (54.7%) and BA (58.4%) was observed to be higher compared to the other two varieties. Additionally, oxygenated monoterpenes appeared to be more abundant than the hydrocarbon analogues for all varieties. Other major monoterpenoids in the samples were α-pinene (2.8–4.0%), β-myrcene (1.5–2.2%), β-phellandrene (5.0–7.9%), eucalyptol (2.3–4.1%), β-citral (5.6–8.1%; or neral), and geraniol (1.8–5.0%). Borneol (2.7%) was found to increase at least three-fold in BA as compared to the other varieties. The concentration of eucalyptol (4.1%) was observed to be about two-fold higher, specifically in BE. Camphor was characteristic particularly to BA (0.1%) and BE (0.1%). Camphene was quantitatively higher in BA (10.5%) and BE (9.4%) as opposed to the other two varieties (7.2% in both CH and SA). Having a strong camphoraceous odor [21,22], it is expected that borneol, eucalyptol, camphor, and camphene possibly contributed to the distinctive flavor and acclaimed medicinal effects of both BE and BA, differentiating them from CH and SA. Meanwhile, CH (6.4%) and BA (2.3%) showed a high relative amount of geranyl acetate in comparison to BE (0.2%) and SA (0.2%), which appeared to be a compound characteristic of Japanese fresh ginger [21].

The sesquiterpenic profile revealed a major contribution of its sesquiterpenes analogues, with SA oil having the highest relative amount (35.6%). The major sesquiterpenes were α-curcumene (2.2–4.5%), zingiberene (7.9–14.0%), β-bisabolene (4.6–8.4%), and β-sesquiphellandrene (3.2–5.2%) for all samples. The concentration of zingiberene was much lower in BE (7.9%) than the other varieties (12.1%, 14.0%, and 10.8% for CH, SA, and BA, respectively). It is noted that this compound constituted the highest in the total composition of CH, SA, and BA. δ-Elemene was found at the low concentrations of 0.3% and 0.1%, in CH and SA, respectively, while it was not detected in BE and BA. Farnesol was present in CH at a low concentration of 0.1% and was absent in the other three varieties. Produced by dephosphorylation of farnesyl-PP, farnesol has been reported to play a critical role in the antiproliferation and apoptosis of various tumor cells [26]. Non-terpenic constituents only accounted for 0.7–2.7% of the total compounds, with the highest relative content present in BE. 2-Nonanol, an acyclic alcohol, was found in BA at only 0.6%; meanwhile, it was absent in the oils from all the other varieties.

*Z. officinale* essential oil was reportedly composed of a relatively high amount of sesquiterpenes and a low concentration of monoterpenes, and this is the case typically associated with Eastern Asian origin [27,28,29,30,31,32,33,34,35]. However, a similarly large number of studies have detailed the preponderance of monoterpenoids from various other locations [28,32,35,36,37,38,39,40,41]. Most of the monoterpenoid-rich oils from previous studies were characterized by significantly high amounts of α- and β-citral. For instance, Wohlmuth et al. reported the amount of citral from Australian-grown *Z. officinale* oils to be in the range of 28.1–70.8% [40]. In a study by Vairappan et al., the content of citral (25.2–28.3%) in the hydro-distilled oil varieties from Malaysia was significantly high [38]. The result did not agree well with the current finding, where the relative amount was comparatively lower (12.9–18.1%) [42]. There are also a few studies where the occurrence of citral is lower and other compounds predominate, similar to the current finding. Mollenbeck et al. found that the *Z. officinale* oil from Madagascar contained camphene (30.8%) as the major compound, while citral was present in a comparatively lower concentration (13.9%) [36]. Nandi et al. reported α-citral concentration to be 7.6–9.8% in the monoterpenoid-rich oils from China and Bangladesh, while β-citral was absent in both oils [28]. The low amounts of citral in *Z. officinale* essential oil have been associated with its loss during drying processes, immaturity of the harvested rhizome, the long storage period before analysis, and changes in climatic conditions during cultivation [37,40]. Other factors that may influence the chemical variation in *Z. officinale* oils include geographical origins, agricultural practices, soil qualities, methods of extraction, and genetics, to adapt to the diverse environmental surroundings [40,43]. These findings demonstrated that these variations may impart significant effects on the flavor and aroma qualities of different varieties of ginger.

### 2.2. Discrimination of Z. officinale Oil Varieties via Principal Component Analysis (PCA)

**Figure 2:**
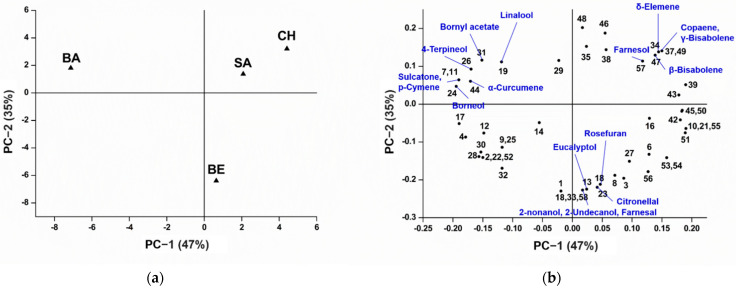
Principal component analysis of 58 secondary compounds in the analyzed *Z. officinale* oils. (**a**) Score plot and (**b**) loading plot. *Z. officinale* varieties: Bentong (BE), Cameron Highlands (CH), Sabah (SA), Bara (BA). The peak numbering refers to Table S1.

Principal component analysis (PCA) was applied to selected secondary compounds to identify differences and inter-relationships between metabolic profiles of the analyzed *Z. officinale* varieties. The score and loading plots in Figure 2 represented 82% of the total data variance (PC −1, 47%, and PC −2, 35%). The score plot clearly indicated that all four *Z. officinale* varieties were well-segregated from each other (Figure 2a), which can be explained in terms of the loading plots of the components (Figure 2b). PC −1 separated BA from SA, CH, and BE. Positioned at the farthest upper left quadrant with the lowest PC −1 score, the segregation of BA can be characterized by the high amounts of sulcatone (0.5%), p-cymene (0.2%), linalool (1.2%), borneol (2.7%), 4-terpineol (0.3%), bornyl acetate (0.6%), and α-curcumene (4.5%). PC −2 separated CH and SA from BE. CH and SA were located near to each other in the same quadrant (positive PC −2 axis), suggesting similarity in terms of secondary compounds and their relative quantity. CH and SA can be segregated from the other varieties through the presence of δ-elemene (0.3%), copaene (0.3%), β-bisabolene (7.4–8.4%), γ-bisabolene (0.2%), and farnesol (0.1%) as the primary loading metabolites. BE, situated near the negative PC −2 axis, contained eucalyptol (4.1%), 2-nonanol (0.6%), 2-undecanol (0.2%), farnesal (0.2%), rosefuran (0.3%), and citronellal (0.8%) as the main discriminatory components separating that variety from the others. Even though most of the principal explanatory variables in the four *Z. officinale* oils contribute to a minor degree to each of their total relative contents, they were proven to have a noticeable influence in distinguishing between different varieties. However, the need for more studies incorporating larger sets of samples for each variety is preferred to confirm their class attributes and chemical resemblance.

### 2.3. Antiproliferative Evaluation

**Figure 3:**
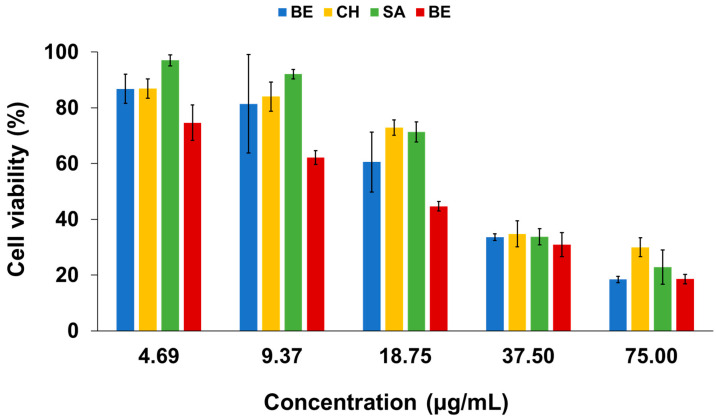
Antiproliferative activity of the four varieties of *Z. officinale* essential oils against the HeLa cervical cancer cell line. *Z. officinale* varieties: Bentong (BE), Cameron Highlands (CH), Sabah (SA), and Bara (BA). Values are means of three replicates ± standard deviation.

The cytotoxicity of the four varieties of *Z. officinale* essential oils was evaluated against the human adenocarcinoma cervical cancer (HeLa) cell line using the 3-(4,5-dimethylthiazol-2-yl)-2,5-diphenyltetrazolium bromide (MTT) assay. All essential oil samples displayed significant cell proliferation activity, in which the growth of the HeLa cells declined in a dose-dependent manner (Figure 3). This result is in agreement with a study reported by Panyajai et al., where the oil of *Zingiber ottensii* also showed dose-dependent anticancer activity against the HeLa cell line [44]. The highest antiproliferative activity was shown by BA oil (IC_50_ = 23.8 μg/mL), followed by BE (IC_50_ = 35.3 μg/mL), SA (IC_50_ = 41.3 μg/mL), and CH (IC_50_ = 42.5 μg/mL). These results were significantly higher as compared to the *Z. officinale* oil from other locations. For example, Santos et al. evaluated the antiproliferative activity of the oil from Southern Brazil against the HeLa human cervical cancer cell line with the IC_50_ value of 141.4 μg/mL [45]. Lee reported the IC_50_ value of Korean ginger oil to be 60.6 μg/mL [46].

Interestingly, these data demonstrate that phenolic compounds (e.g., 6-gingerol, shogaol, etc.) in ginger are not the only bioactive molecules responsible for conferring the antiproliferative effects of cancer cells (typically with IC_50_ values in the range of 16.0–253.4 μg/mL) [47,48,49,50,51]. It is likely that the encouraging antiproliferative activity of ginger is also attributable to the terpenic components (or synergistic effects of the components) present in the essential oils. For instance, α-zingiberene (or zingiberene) was found to induce DNA fragmentation, increase the sub-diploid cell population, and activate caspase-3, leading to the pathway for cancer apoptosis [46]. Likewise, the inhibition of Ishikawa and ECC-1 endometrial cell proliferation were lower when treated with citral (α- and β-) as opposed to treatment with *Z. officinale* oil [15]. The study found that the terpenoids that are present in the oil served as potent anticancer agents that activated the p53 tumor suppressor, which then triggered the apoptotic pathways in endometrial cancer cells. Hachlafi et al. reported that camphene (a major compound in BA) can trigger apoptosis by decreasing pro-caspases 9 and induce the increase of caspases 3 through the activation of poly (ADP-ribose) polymerase cleavage [52]. These findings indicated that the synergism of various secondary compounds plays an important role in inducing cell cycle arrest and apoptosis in different cancer cell lines.

## 3. Materials and Methods

### 3.1. Chemicals and Reagents

Heptane (99%), nonane (99%), undecane (≥99%), dodecane (99%), tridecane (≥99%), tetradecane (≥99%), pentadecane (≥99%), hexadecane, heptadecane (99%), octadecane (99%), nonadecane (99%), eicosane (99%), heneicosane (≥99.5%), docosane (99%), tricosane (99%), tetracosane (99%), pentacosane (99%), hexacosane (99%), octacosane (99%), and triacontane (99%) were purchased from Sigma-Aldrich (Darmstadt, Germany). Octane (98%) and decane (≥99%) were purchased from Sigma-Aldrich (St. Louis, MO, USA). Acetone and hexane were of HPLC grade and purchased from Elite Advanced Materials Sdn. Bhd. (Selangor, Malaysia). Ultrapure water (18.2 MΩ cm^−1^) was purified by a Millipore Milli-Q system (Bedford, MA, USA).

### 3.2. Preparation and Isolation of Essential Oil

Four different varieties of mature *Zingiber officinale* rhizomes (Bentong (BE), Bara (BA), Cameron Highlands (CH), and Sabah (SA)) were collected from local producers in Malaysia. The samples were rinsed with ultra-pure water to remove particulates, air-dried (12 h), and grated into small pieces. Approximately 800 g of the grated samples was subjected to steam distillation for 5 h. The oils were isolated and stored at 4 °C when not in use. Prior to analysis, oil samples were diluted to 3% *v*/*v* with acetone.

### 3.3. Chromatographic Condition

Gas chromatography−mass spectrometry (GC-MS) analyses were conducted on an Agilent Technologies 7890B GC system equipped with a 5977B GC/MSD quadrupole mass spectrometer (Agilent Technologies, Santa Clara, CA, USA), a 7693A autosampler, and a split/split-less inlet. The chromatographic separation was performed using a HP-5ms (5% phenyl-methylpolysiloxane) capillary column of dimensions 30 m × 0.25 mm I.D. × 0.25 μm film thickness (Agilent Technologies, Santa Clara, CA, USA). The chromatographic conditions were: oven temperature program, 40 °C (hold 2 min), then heated at 3 °C min^−1^ to 270 °C; injector temperature, 300 °C; carrier gas, helium (purity of 99.999%) at a flow rate of 1.0 mL min^−1^ (24.02 cm s^−1^); injection volume, 1 µL, and using a split ratio of 20:1. The MS was operated in electron ionization (EI) mode at 70 eV, transfer line temperature of 300 °C, ion source temperature of 230 °C, mass scan range of 45–500 Da, and solvent delay time of 2.6 min.

### 3.4. Data Handling and Statistical Analysis

Data acquisition and processing were performed using Agilent MassHunter Qualitative Analysis 10.0 (Agilent Technologies, Santa Clara, CA, USA). The National Institute of Standards and Technology (NIST) 14 MS spectrum library was used for spectrum searching and identification. Retention indices (RI) values were determined with respect to a C_7_–C_30_ series of n-alkane standards analyzed under the same GC conditions as above, using the Van den Dool and Kratz equation and compared with reported RI values [23,24,53]. The relative concentrations of tentatively identified components were calculated based on the acquired total ion chromatograms and presented as the mean ± standard deviation from three repeated independent experiments. The data were statistically analyzed using PCA (Unscrambler X 10.3; CAMO Software AS, Oslo, Norway) to identify differences for the obtained chemical profiles. All data were presented using Origin 8 (OriginLab Corporation, Northampton, MA, USA) and Excel software (Microsoft Corporation, Washington, DC, USA).

### 3.5. Cell Culture

The human cervical adenocarcinoma (HeLa) cell line was acquired from the American Type Culture Collection (Manassas, VA, USA). The cell was cultured in Dulbecco’s modified Eagle’s medium—high glucose (DMEM, Sigma-Aldrich, St. Louis, MO, USA), supplemented with 10% fetal bovine serum (FBS) and 1% penicillin or streptomycin at 37 °C and 5% CO_2_.

### 3.6. Cell Viability Assay

A total of 3 × 10^3^ cells/well were seeded into a 96-well plate in supplemented DMEM and incubated (5% CO_2_ at 37 °C) for 24 h. The medium was removed and the essential oils, re-suspended in ethanol (Merck, Darmstadt, Germany) at a final concentration of 50 mg/mL, and diluted with supplemented DMEM to afford concentration ranges from 4.69 to 75.00 µg/mL, were added to each well. The final concentration of ethanol in each well was not more than 0.3%. On completion of the 24 h incubation period, the MTT assay, as described by Mossman [25], was performed to assess the cell viability. The optical density (OD) was recorded at 540 nm by using a SkanIT absorbance reader (Thermo Scientific, St. Peters, MO, USA). All experiments were performed in triplicate using media with cells only as a positive control, and only media with no cells as a negative control. The vehicle control was cells and media with 0.3% ethanol.

## 4. Conclusions

This study reported a detailed untargeted characterization of the phytoconstituents of essential oils derived from the rhizomes of four varieties of *Z. officinale* (BE, CH, SA, and BA) sourced in Malaysia, using GC-qMS. Interestingly, the metabolic profiles of the oils revealed differences in terms of their major classes of compounds according to varieties. SA was dominated by sesquiterpenic hydrocarbons, whilst BE, CH, and BA were dominated by oxygenated monoterpenes. α-Citral (9.8%) was identified as the most abundant metabolite in BE, whilst zingiberene (10.8–12.1%) in CH, SA, and BA. PCA successfully classified different *Z. officinale* oils into their corresponding varieties, characterized according to their metabolic compositions. The oils displayed strong antiproliferative activities against the HeLa cell line, in which BA exerted the most significant inhibition (IC_50_ value of 23.8 μg/mL). This study indicated that essential oils extracted from different *Z. officinale* cultivars constitute different phytochemical diversity, and thus confer distinctive flavor characteristics and antiproliferative effects. Moreover, these data also demonstrated the strong antiproliferation potential of ginger essential oils, which can be further explored as a potent alternative for the prevention and treatment of cervical carcinoma.

## Figures and Tables

**Table 1:** Major secondary compounds tentatively identified in different ginger oils using GC−MS. Repeated entries for a given compound correspond to the different ginger varieties.

No	Compound	^a^ T_R_ (min)	CASRN	Molecular Formula	^b^ Class	^c^ Match Factor (Reverse Match Factor)	^d^*m/z* of Significant Ions (Relative Ion Abundance)	RI_ref_	^e^ Ri_cal_ ^f^ (Relative Percentage Abundance, %)			
									BE	CH	SA	BA
1	Pinene, α-	10.0	80-56-8	C_10_H_16_	*MH*	931 (932); 934 (934); 930 (930); 933 (933)	93.1 (100), 91.1 (43.3), 92.1 (37.5); 93.1 (100), 91.1 (43.3), 92.1 (37.5); 93.1 (100), 91.1 (45.3), 92.1 (37.8); 93.1 (100), 91.1 (42.4), 92.1 (37.4)	931	931 (4.0 ± 0.71)	931 (3.3 ± 0.83)	931 (3.0 ± 0.04)	931 (2.8 ± 0.37)
												
2	Camphene	10.7	79-92-5	C_10_H_16_	*MH*	958 (967); 959 (966); 958 (965); 959 (967)	93.1 (100), 121.2 (74.9), 79.1 (36.2); 93.1 (100), 121.2 (76.7), 79.1 (35.9); 93.1 (100), 121.1 (72.6), 91.1 (36.2); 93.1 (100), 121.2 (76.8), 79.1 (36.2)	946	946 (9.4 ± 1.71)	946 (7.2 ± 1.75)	945 (7.2 ± 0.10)	946 (10.5 ± 1.45)
												
3	Myrcene, β-	12.7	123-35-3	C_10_H_16_	*MH*	941 (949); 930 (938); 925 (933); 935 (944)	93.1 (100), 69.1 (65.1), 91.1 (23.0); 93.1 (100), 69.1 (64.0), 91.1 (23.0); 93.1 (100), 69.1 (62.9), 91.1 (24.4); 93.1 (100), 69.1 (64.5), 91.1 (22.8)	991	992 (2.2 ± 0.39)	992 (1.8 ± 0.48)	991 (1.5 ± 0.02)	991 (1.5 ± 0.18)
												
4	Phellandrene, β-	14.4	555-10-2	C_10_H_16_	*MH*	867 (904); 857 (884); 901 (904); 883 (895)	93.1 (100), 91.1 (44.1), 77.1 (33.0); 93.1 (100), 91.1 (43.2), 77.1 (32.3); 93.1 (100), 91.1 (43.2), 77.1 (32.3); 93.1 (100), 91.1 (44.7), 77.1 (33.2)	1028	1028 (7.2 ± 1.22)	1028 (5.0 ± 1.25)	1028 (7.4 ± 0.08)	1028 (7.9 ± 1.01)
												
5	Eucalyptol	14.5	470-82-6	C_10_H_18_O	*MO*	940 (940); 931 (931); 917 (917); 934 (934)	81.1 (100), 108.1 (92.7), 111.1 (80.3); 81.1 (100), 108.1 (94.4), 154.2 (80.9); 81.1 (100), 108.1 (97.4), 111.1 (85.6); 81.1 (100), 108.1 (92.8), 154.2 (79.7)	1029	1029 (4.1 ± 0.68)	1029 (2.4 ± 0.64)	1028 (2.3 ± 0.038)	1029 (2.3 ± 0.31)
												
6	Linalool	17.8	78-70-6	C_10_H_18_O	*OM*	887 (902); 892 (900); 891 (901); 864 (880)	71.1 (100), 93.1 (90.0), 69.1 (62.5); 71.1 (100), 93.1 (90.4), 69.1 (63.3); 71.1 (100), 93.1 (94.2), 69.1 (58.1); 71.1 (100), 93.1 (88.1), 69.1 (60.4)	1100	1100 (0.7 ± 0.09)	1100 (1.0 ± 0.51)	1100 (0.6 ± 0.008)	1100 (1.2 ± 0.14)
												
7	Borneol	20.8	507-70-0	C_10_H_18_O	*OM*	923 (923); 917 (917); 910 (910); 940 (940)	95.1 (100), 110.2 (20.6), 93.1 (9.1); 95.1 (100), 110.1 (20.7), 93.1 (9.0); 95.1 (100), 110.1 (20.2), 67.1 (9.8); 95.1 (100), 110.2 (21.1), 7.1 (9.6)	1163	1163 (0.8 ± 0.09)	1163 (0.6 ± 0.28)	1163 (0.7 ± 0.01)	1163 (2.7 ± 0.30)
												
8	Citral, β-	24.6	106-26-3	C_10_H_16_O	*MA*	929 (929); 936 (936); 926 (926); 936 (937)	69.1 (100), 94.1 (37.0), 109.1 (36.2); 69.1 (100), 94.1 (36.6), 109.1 (35.4); 69.1 (100), 109.1 (40.0), 94.1 (37.8); 69.1 (100), 94.1 (36.9), 109.1 (35.9)	1244	1245 (8.0 ± 0.98)	1244 (5.6 ± 0.53)	1244 (6.8 ± 0.03)	1244 (8.1 ± 0.92)
												
9	Geraniol	25.3	106-24-1	C_10_H_18_O	*OM*	930 (930); 929 (929); 924 (924); 937 (937)	69.1 (100), 68.1 (20.0), 93.1 (17.3); 69.1 (100), 68.1 (19.9), 93.1 (18.2); 69.1 (100), 68.1 (20.0), 93.1 (19.2); 69.1 (100), 68.1 (19.9), 93.1 (14.6)	1259	1259 (2.6 ± 0.32)	1260 (5.0 ± 0.57)	1258 (1.8 ± 0.008)	1259 (4.2 ± 0.43)
												
10	Citral, α-	26.0	141-27-5	C_10_H_16_O	*MA*	942 (942); 943 (943); 924 (924); 939 (939)	69.1 (100), 84.1 (29.4), 94.1 (19.1); 69.1 (100), 84.1 (29.4), 94.1 (19.1); 69.1 (100), 84.1 (28.2), 94.1 (19.2); 69.1 (100), 84.1 (29.6), 94.1 (19.3)	1268	1276 (9.8 ± 2.52)	1275 (7.3 ± 0.71)	1275 (9.0 ± 0.01)	1268 (10.0 ± 1.11)
												
11	Geranyl acetate	30.8	105-87-3	C_12_H_20_O_2_	*MAc*	893 (905); 934 (937); 881 (899); 941 (942)	69.1 (100), 93.1 (46.9), 68.1 (41.6); 69.1 (100), 68.1 (38.8), 93.1 (34.3); 69.1 (100), 93.1 (52.4), 68.1 (37.7); 69.1 (100), 68.1 (38.6), 93.1 (34.4)	1385	1385 (0.2 ± 0.05)	1388 (6.4 ± 0.53)	1385 (0.2 ± 0.001)	1386 (2.3 ± 0.58)
												
12	Germacrene D	34.7	23986-74-5	C_15_H_24_	*SH*	912 (933); 919 (936); 916 (934); 863 (891)	161.2 (100), 105.1 (54.1), 91.1 (47.0); 161.2 (100), 105.1 (54.5), 91.1 (46.5); 161.2 (100), 105.1 (54.9), 91.1 (48.1); 161.2 (100), 119.1 (86.4), 105.1 (72.7)	1480	1480 (0.9 ± 0.15)	1480 (1.2 ± 0.50)	1480 (1.5 ± 0.02)	1480 (0.3 ± 0.04)
												
13	Curcumene, α-	34.9	644-30-4	C_15_H_22_	*SH*	917 (936); 909 (937); 908 (932); 932 (940)	119.1 (100), 132.1 (87.6), 105.1 (52.5); 119.1 (100), 132.1 (86.7), 105.1 (53.1); 119.1 (100), 132.1 (87.5), 105.1 (53.9); 119.1 (100), 132.1 (88.8), 105.1 (50.3)	1484	1484 (2.6 ± 0.40)	1483 (2.2 ± 0.22)	1484 (3.6 ± 0.06)	1484 (4.5 ± 0.85)
												
14	Zingiberene	35.5	495-60-3	C_15_H_24_	*SH*	861 (886); 902 (955); 896 (951); 898 (950)	119.1 (100), 93.1 (82.9), 91.1 (46.8); 119.1 (100), 93.1 (84.4), 91.1 (42.8); 119.1 (100), 93.1 (82.9), 91.1 (46.7); 119.1 (100), 93.1 (84.3), 91.1 (43.7)	1499	1499 (7.9 ± 1.13)	1498 (12.1 ± 1.01)	1497 (14.0 ± 1.29)	1499 (10.8 ± 1.86)
												
15	Bisabolene, β-	36.0	495-61-4	C_15_H_24_	*SH*	890 (899); 942 (948); 936 (942); 886 (886)	93.1 (100), 69.1 (56.3), 107.1 (48.1); 93.1 (100), 107.1 (52.6), 119.1 (47.2); 93.1 (100), 107.1 (54.9), 91.1 (46.5); 93.1 (100), 69.1 (66.1), 107.1 (44.0)	1510	1510 (4.6 ± 0.70)	1510 (7.4 ± 0.60)	1510 (8.4 ± 0.16)	1510 (4.6 ± 0.91)
												
16	Sesquiphellandrene, β-	36.6	20307-83-9	C_15_H_24_	*SH*	906 (917); 936 (925); 899 (910); 912 (936)	69.1 (100), 93.1 (68.4), 91.1 (64.8); 69.1 (100), 93.1 (68.0), 91.1 (61.1); 69.1 (100), 93.1 (68.0), 91.1 (61.1); 69.1 (100), 93.1 (67.6), 91.1 (60.9)	1525	1525 (3.2 ± 0.51)	1526 (4.6 ± 0.47)	1526 (5.2 ± 0.08)	1526 (4.5 ± 0.97)
												
17	Elemol	37.5	21657-90-9	C_15_H_26_O	*OS*	926 (929); 926 (929); 941 (946); 918 (921)	93.1 (100), 59.1 (98.7), 161.2 (80.1); 93.1 (100), 59.1 (96.9), 161.2 (81.9); 93.1 (100), 59.1 (91.3), 161.2 (85.4); 93.1 (100), 59.1 (97.7), 161.2 (80.8)	1549	1549 (0.9 ± 0.12)	1549 (1.2 ± 0.12)	1549 (0.7 ± 0.01)	1549 (0.3 ± 0.08)

^a^ Retention time of eluted compounds. ^b^ Class of chemical compounds: *MH* monoterpenic hydrocarbon, *MO* monoterpenic oxide, *MA* monoterpenic aldehyde, *OM* monoterpenic alcohol, *MAc* monoterpenic acetate, *SH* sesquiterpenic hydrocarbon, *OS* sesquiterpenic alcohol. ^c^ Matching scores of compounds reported ≥80% based on mass spectra in NIST library database and in the order of Bentong (BE), Cameron Highlands (CH), Sabah (SA) and Bara (BA) gingers. ^d^ Fragmentation patterns reported in order of BE, CH, SA and BA. ^e^ Retention index (RI) values calculated using Van Den Dool and Kratz equation with the reference to reported RI values [23,24,25] within the range of ±10. ^f^ Relative percentage abundance calculated on the basis of TIC (Total Ion Chromatogram) area as the percentage of total TIC area.

## Data Availability

Data are available upon request.

## Supplementary Materials

The following supporting information can be downloaded at: https://www.mdpi.com/article/10.3390/plants11101280/s1, Table S1: Secondary compounds analyzed and identified in different ginger oil varieties using GC−MS. Repeated entries for a given compound correspond to different ginger varieties, Figure S1: Total ion chromatograms (TICs) of the volatile profile of *Z. officinale* essential oil varieties. (a) Bentong, BE; (b) Cameron Highlands, CH; (c) Sabah, SA; and (d) Bara, BA. The peak numbering refers to Table S1.
