# Supplementary material for: Phytochemical Constituents and Antiproliferative Activities of Essential Oils from Four Varieties of Malaysian Zingiber officinale Roscoe against Human Cervical Cancer Cell Line
Source: Plants (Basel). 2022 May 10;11(10):1280. doi: 10.3390/plants11101280 (PMC9143566; doi:10.3390/plants11101280)
Supplement: Supplementary file 1 [file plants-11-01280-s001.zip › plants-1718640-supplementary.pdf]

Phytochemical Constituents and Antiproliferative Activities of Essential Oils from Four Varieties of Malaysian *Zingiber officinale* Roscoe against Human Cervical Cancer Cell Line

Atiqah Zaid [1], Xue Rou Haw [1], Huda Hisham Alkatib [2], Sreenivasan Sasidharan [2], Philip J. Marriott [3] and Yong Foo Wong [1,*]

[1] Centre for Research on Multidimensional Separation Science, School of Chemical Sciences, Universiti Sains Malaysia, Penang 11800, Malaysia; atiqahzaid@student.usm.my (A.Z.); xuerouhaw306@gmail.com (X.R.H.)
[2] Institute for Research in Molecular Medicine (INFORMM), Universiti Sains Malaysia, Penang 11800, Malaysia; huda.alkatib22@gmail.com (H.H.A.); srisasidharan@usm.my (S.S.)
[3] Australian Centre for Research on Separation Science, School of Chemistry, Monash University, Wellington Road, Clayton, Melbourne, VIC 3800, Australia; philip.marriott@monash.edu
* Correspondence: wongyongfoo@usm.my; Tel.: +604 653 4031

Supplementary material

Table S1 Secondary compounds tentatively identified in different ginger oils using GC−MS. Repeated entries for a given compound correspond to the different ginger varieties.

No	Compound	[a]T_R (min)	CASRN	Molecular formula	[b]Class	[c]Match Factor (Reverse Match Factor)	[d]m/z of significant ions (relative ion abundance)	RI_{ref}	[e]Ri_{cal} [f](relative percentage abundance, %)			
									**BE**	**CH**	**SA**	**BA**
**1**	2-Heptanol	8.7	543-49-7	$C_7H_{16}O$	*OA*	922 (923);<br>920 (924);<br>911 (914)	45.1(100), 55.1 (21.9), 83.1 (14.8);<br>45.1(100), 55.1 (25.8), 83.1 (16.4);<br>45.1(100), 55.1 (23.4), 83.1 (15.2)	**901**	901 (0.4 ± 0.065)	[g]NA	901 (0.1 ± 0.002)	901 (0.1 ± 0.016)
**2**	Tricyclene	9.5	508-32-7	$C_{10}H_{16}$	*MH*	913 (913);<br>912 (912);<br>909 (909);<br>917 (917)	93.1 (100), 91.1 (29.8), 121.1 (24.2);<br>93.1 (100), 91.1 (29.5), 121.1 (24.6);<br>93.1 (100), 91.1 (30.9), 121.1 (22.9);<br>93.1 (100), 91.1 (29.9), 121.1 (24.7)	**919**	918 (0.3 ± 0.046)	919 (0.2 ± 0.046)	918 (0.2 ± 0.001)	918 (0.3 ± 0.036)
**3**	Pinene, α-	10.0	80-56-8	$C_{10}H_{16}$	*MH*	931 (932);<br>934 (934);<br>930 (930);<br>933 (933)	93.1 (100), 91.1 (43.3), 92.1 (37.5);<br>93.1 (100), 91.1 (43.3), 92.1 (37.5);<br>93.1 (100), 91.1 (45.3), 92.1 (37.8);<br>93.1 (100), 91.1 (42.4), 92.1 (37.4)	**931**	931 (4.0 ± 0.714)	931 (3.3 ± 0.831)	931 (3.0 ± 0.035)	931 (2.8 ± 0.371)
**4**	Camphene	10.7	79-92-5	$C_{10}H_{16}$	*MH*	958 (967);<br>959 (966);<br>958 (965);<br>959 (967)	93.1 (100), 121.2 (74.9), 79.1 (36.2);<br>93.1 (100), 121.2 (76.7), 79.1 (35.9);<br>93.1 (100), 121.1 (72.6), 91.1 (36.2);<br>93.1 (100), 121.2 (76.8), 79.1 (36.2)	**946**	946 (9.4 ± 1.708)	946 (7.2 ± 1.748)	945 (7.2 ± 0.101)	946 (10.5 ± 1.452)
**5**	Sabinene	11.8	3387-41-5	$C_{10}H_{16}$	*MH*	914 (929);<br>922 (928);<br>907 (914);<br>901 (909)	93.1 (100), 91.1 (40.2), 77.1 (33.5);<br>93.1 (100), 91.1 (42.6), 77.1 (33.3);<br>93.1 (100), 91.1 (44.2), 77.1 (34.7);<br>93.1 (100), 91.1 (43.0), 77.1 (31.4)	**971**	970 (0.1 ± 0.022)	971 (0.1 ± 0.027)	971 (0.1 ± 0.001)	971 (0.1 ± 0.013)
**6**	Pinene, β-	11.9	127-91-3	$C_{10}H_{16}$	*MH*	906 (906);<br>910 (910);<br>893 (893);<br>900 (900)	93.1 (100), 69.1 (28.2), 91.1 (27.9);<br>93.1 (100), 91.1 (27.4), 69.1 (27.2);<br>93.1 (100), 91.1 (29.6), 69.1 (26.0);<br>93.1 (100), 69.1 (27.4), 91.1 (27.3)	**973**	973 (0.8 ± 0.139)	973 (0.7 ± 0.180)	973 (0.4 ± 0.004)	973 (0.3 ± 0.035)
**7**	Sulcatone	12.5	110-93-0	$C_8H_{14}O$	*AK*	906 (914);<br>892 (899);<br>863 (866);<br>912 (919)	108.1 (100), 69.1 (74.8), 55.1 (69.7);<br>108.1 (100), 69.1 (73.0), 55.1 (68.1);<br>108.1 (100), 69.1 (70.5), 55.1 (68.5);<br>108.1 (100), 69.1 (73.3), 55.1 (67.2)	**987**	987 (0.3 ± 0.065)	987 (0.1 ± 0.027)	987 (0.1 ± 0.002)	987 (0.5 ± 0.062)

Table S1 (continued)

8	Myrcene, β-	12.7	123-35-3	$C_{10}H_{16}$	*MH*	941 (949); 930 (938); 925 (933); 935 (944)	93.1 (100), 69.1 (65.1), 91.1 (23.0); 93.1 (100), 69.1 (64.0), 91.1 (23.0); 93.1 (100), 69.1 (62.9), 91.1 (24.4); 93.1 (100), 69.1 (64.5), 91.1 (22.8)	991	992 (2.2 ± 0.394)	992 (1.8 ± 0.483)	991 (1.5 ± 0.022)	991 (1.5 ± 0.182)
**9**	Phellandrene, α-	13.2	99-83-2	$C_{10}H_{16}$	*MH*	845 (921); 856 (922); 876 (912); 888 (923)	93.1 (100), 91.1 (55.7), 77.1 (33.4); 93.1 (100), 91.1 (55.1), 77.1 (32.6); 93.1 (100), 91.1 (57.7), 77.1 (34.2); 93.1 (100), 91.1 (55.4), 77.1 (33.1)	**1002**	1002 (0.6 ± 0.102)	1002 (0.5 ± 0.118)	1002 (0.6 ± 0.009)	1002 (0.6 ± 0.073)
**10**	3-Carene	13.5	13466-78-9	$C_{10}H_{16}$	*MH*	921 (928); 920 (924); 919 (923); 937 (938)	93.1 (100), 91.1 (46.1), 77.1 (30.6); 93.1 (100), 91.1 (48.1), 77.1 (30.7); 93.1 (100), 91.1 (49.7), 77.1 (35.3); 93.1 (100), 91.1 (49.3), 77.1 (31.9)	**1008**	1008 (0.1 ± 0.021)	1008 (0.1 ± 0.023)	1008 (0.1 ± 0.032)	1008 (0.06 ± 0.009)
**11**	Cymene, p-	14.2	99-87-6	$C_{10}H_{14}$	*MH*	884 (888); 940 (945); 874 (876); 890 (891)	119.1 (100), 91.1 (22.3), 134.1 (20.9); 119.1 (100), 134.1 (27.4), 91.1 (18.8); 119.1 (100), 134.1 (28.0), 91.1 (17.7); 119.1 (100), 134.1 (28.0), 91.1 (19.7)	**1023**	1023 (0.1 ± 0.025)	1023 (0.1 ± 0.015)	1023 (0.1 ± 0.004)	1023 (0.2 ± 0.025)
**12**	Phellandrene, β-	14.4	555-10-2	$C_{10}H_{16}$	*MH*	867 (904); 857 (884); 901 (904); 883 (895)	93.1 (100), 91.1 (44.1), 77.1 (33.0); 93.1 (100), 91.1 (43.2), 77.1 (32.3); 93.1 (100), 91.1 (43.2), 77.1 (32.3); 93.1 (100), 91.1 (44.7), 77.1 (33.2)	**1028**	1028 (7.2 ± 1.217)	1028 (5.0 ± 1.251)	1028 (7.4 ± 0.079)	1028 (7.9 ± 1.012)
**13**	Eucalyptol	14.5	470-82-6	$C_{10}H_{18}O$	*MO*	940 (940); 931 (931); 917 (917); 934 (934)	81.1 (100), 108.1 (92.7), 111.1 (80.3); 81.1 (100), 108.1 (94.4), 154.2 (80.9); 81.1 (100), 108.1 (97.4), 111.1 (85.6); 81.1 (100), 108.1 (92.8), 154.2 (79.7)	**1029**	1029 (4.1 ± 0.675)	1029 (2.4 ± 0.642)	1028 (2.3 ± 0.038)	1029 (2.3 ± 0.314)
**14**	2-Heptanol, acetate	15.2	5921-82-4	$C_9H_{18}O_2$	*AE*	926 (927); 887 (892); 932 (932)	87.1 (100), 56.1 (45.9), 98.1 (41.4); 87.1 (100), 56.1 (42.2), 98.1 (40.5); 87.1 (100), 56.1 (41.3), 98.2 (38.5)	**1054**	1044 (0.1 ± 0.010)	1044 (0.1 ± 0.040)	NA	1044 (0.1 ± 0.013)

Table S1 (continued)

**15**	Terpinene, γ-	15.8	99-85-4	$C_{10}H_{16}$	*MH*	817 (912);<br>877 (899);<br>813 (870);<br>848 (889)	93.1 (100), 91.1 (49.1), 136.2 (35.4);<br>93.1 (100), 91.1 (53.3), 136.2 (43.0);<br>93.1 (100), 91.1 (47.6), 136.2 (45.5);<br>93.1 (100), 91.1 (49.9), 136.2 (36.3)	**1057**	1057<br>(0.1 ±<br>0.021)	1057<br>(0.1 ±<br>0.021)	1057<br>(0.1 ±<br>0.001)	1057<br>(0.1 ±<br>0.010)
16	Terpinolene	17.2	586-62-9	$C_{10}H_{16}$	*MH*	902 (910);<br>915 (923);<br>875 (876);<br>896 (907)	121.1 (100), 93.1 (94.7), 136.2 (87.6);<br>121.1 (100), 93.1 (92.8), 136.2 (88.2);<br>121.1 (100), 93.1 (91.8), 136.2 (87.7);<br>121.1 (100), 93.1 (92.6), 136.2 (87.8)	1085	1086<br>(0.6 ±<br>0.092)	1086<br>(0.7 ±<br>0.331)	1086<br>(0.4 ±<br>0.005)	1086<br>(0.4 ±<br>0.065)
**17**	2-Nonanone	17.5	821-55-6	$C_9H_{18}O$	*AK*	873 (887);<br>879 (910);<br>818 (853);<br>924 (928)	58.1 (100), 71.1 (26.0), 59.1 (22.8);<br>58.1 (100), 71.1 (25.7), 57.1 (21.6);<br>58.1 (100), 71.1 (24.3), 57.1 (23.7);<br>58.1 (100), 71.1 (26.0), 57.1 (23.2)	**1092**	1092<br>(0.3 ±<br>0.035)	1092<br>(0.1 ±<br>0.046)	1092<br>(0.1 ±<br>0.002)	1092<br>(0.5 ±<br>0.055)
**18**	Rosefuran	17.7	15186-51-3	$C_{10}H_{14}O$	*MO*	904 (906);<br>920 (924);<br>873 (874);<br>914 (919)	150.2 (100), 135.1 (89.6), 91.1 (39.7);<br>150.1 (100), 135.1 (80.9), 91.1 (41.7);<br>150.1 (100), 135.1 (92.1), 91.1 (41.1);<br>150.2 (100), 135.1 (93.2), 91.1 (42.5)	**1095**	1097<br>(0.3 ±<br>0.036)	1097<br>(0.1 ±<br>0.003)	1097<br>(0.2 ±<br>0.008)	1097<br>(0.1 ±<br>0.011)
**19**	Linalool	17.8	78-70-6	$C_{10}H_{18}O$	*OM*	887 (902);<br>892 (900);<br>891 (901);<br>864 (880)	71.1 (100), 93.1 (90.0), 69.1 (62.5);<br>71.1 (100), 93.1 (90.4), 69.1 (63.3);<br>71.1 (100), 93.1 (94.2), 69.1 (58.1);<br>71.1 (100), 93.1 (88.1), 69.1 (60.4)	**1100**	1100<br>(0.7 ±<br>0.090)	1100<br>(1.0 ±<br>0.507)	1100<br>(0.6 ±<br>0.008)	1100<br>(1.2 ±<br>0.125)
**20**	2-Nonanol	17.9	628-99-9	$C_9H_{20}O$	*OA*	866 (920)	45.1 (100), 69.1 (35.2), 55.1 (19.1)	**1100**	1101<br>(0.6 ±<br>0.092)	NA	NA	NA
**21**	1,3,7-Nonatriene,<br>4,8-dimethyl-	18.7	19945-61-0	$C_{11}H_{18}$	*AH*	893 (912);<br>895 (912);<br>892 (900)	69.1 (100), 81.1 (19.0), 79.1 (16.5);<br>69.1 (100), 81.1 (18.5), 79.1 (16.3);<br>69.1 (100), 81.1 (20.7), 79.0 (20.2)	**1118**	1117<br>(0.1 ±<br>0.013)	1117<br>(0.1 ±<br>0.058)	1117<br>(0.1 ±<br>0.005)	NA
**22**	Camphor	19.8	76-22-2	$C_{10}H_{16}O$	*MK*	941 (941);<br>951 (958)	95.1 (100), 81.1 (66.7), 108.1 (47.4);<br>95.1 (100), 81.1 (65.1), 108.1 (40.9)	**1142**	1141<br>(0.1 ±<br>0.008)	NA	NA	1141<br>(0.1 ±<br>0.012)

Table S1 (continued)

**23**	Citronellal	20.4	2385-77-5	$C_{10}H_{18}O$	*MA*	918 (918); 913 (913); 922 (923); 890 (890)	69,1 (100), 95.1 (77.4), 55.1 (45.9); 69,1 (100), 95.1 (86.2), 55.1 (46.9); 69,1 (100), 95.1 (86.8), 55.1 (46.7); 95.1 (100), 69.1 (82.7), 55.1 (40.6)	**1152**	1154 (0.8 ± 0.081)	1153 (0.3 ± 0.140)	1153 (0.5 ± 0.003)	1154 (0.3 ± 0.040)
**24**	Borneol	20.8	507-70-0	$C_{10}H_{18}O$	*OM*	923 (923); 917 (917); 910 (910); 940 (940)	95.1 (100), 110.2 (20.6), 93.1 (9.1); 95.1 (100), 110.1 (20.7), 93.1 (9.0); 95.1 (100), 110.1 (20.2), 67.1 (9.8); 95.1 (100), 110.2 (21.1), 7.1 (9.6)	**1163**	1163 (0.8 ± 0.094)	1163 (0.6 ± 0.282)	1163 (0.7 ± 0.012)	1163 (2.7 ± 0.302)
25	Iso-citral	20.9	72203-97-5	$C_{10}H_{16}O$	*MA*	986 (907); 925 (926); 875 (876); 889 (890)	109.1 (100), 81.1 (96.6), 67.1 (94.3); 109.1 (100), 81.1 (93.2), 94,1 (90.6); 109.1 (100), 81.1 (96.6), 67.1 (93.3); 109.1 (100), 94.1 (93.6), 81.1 (92.6)	**1160**	1165 (0.5 ± 0.054)	1165 (0.4 ± 0.244)	1165 (0.5 ± 0.009)	1165 (0.5 ± 0.059)
**26**	4-Terpineol	21.4	562-74-3	$C_{10}H_{18}O$	*OM*	803 (834); 852 (876); 800 (825); 801 (836)	71.1 (100), 95.1 (70.1),111.1 (65.6); 71.1 (100), 93.1 (70.7), 111.1 (69.9); 71.1 (100), 111.1 (69.3), 93.1 (65.6); 71.1 (100), 95.1 (92.1), 111.1 (66.0)	**1175**	1175 (0.1 ± 0.015)	1175 (0.1 ± 0.039)	1175 (0.2 ± 0.09)	1175 (0.3 ± 0.031)
**27**	Terpineol, α-	22.1	98-55-5	$C_{10}H_{18}O$	*OM*	907 (907); 900 (900); 882 (882); 899 (899)	59.1 (100), 93.1 (82.2), 121.1 (75.7); 59.1 (100), 93.1 (83.8), 121.1 (78.0); 59.1 (100), 93.1 (88.3), 121.1 (85.2); 59.1 (100), 93.1 (81.7), 121.1 (76.1)	**1189**	1189 (0.9 ± 0.114)	1189 (0.8 ± 0.242)	1189 (0.6 ± 0.006)	1189 (0.6 ± 0.070)
**28**	Citral, β-	24.6	106-26-3	$C_{10}H_{16}O$	*MA*	929 (929); 936 (936); 926 (926); 936 (937)	69.1 (100), 94.1 (37.0), 109.1 (36.2); 69.1 (100), 94.1 (36.6), 109.1 (35.4); 69.1 (100), 109.1 (40.0), 94.1 (37.8); 69.1 (100), 94.1 (36.9), 109.1 (35.9)	**1244**	1245 (8.0 ± 0.981)	1244 (5.6 ± 0.528)	1244 (6.8 ± 0.030)	1244 (8.1 ± 0.922)
**29**	Geraniol	25.3	106-24-1	$C_{10}H_{18}O$	*OM*	930 (930); 929 (929); 924 (924); 937 (937)	69.1 (100), 68.1 (20.0), 93.1 (17.3); 69.1 (100), 68.1 (19.9), 93.1 (18.2); 69.1 (100), 68.1 (20.0), 93.1 (19.2); 69.1 (100), 68.1 (19.9), 93.1 (14.6)	**1259**	1259 (2.6 ± 0.319)	1260 (5.0 ± 0.568)	1258 (1.8 ± 0.008)	1259 (4.2 ± 0.428)

Table S1 (continued)

**31**	Bornyl acetate	26.5	5655-61-8	$C_{12}H_{20}O_2$	*MAc*	916 (917);<br>912 (912);<br>910 (910);<br>917 (917)	95.1 (100), 93.1 (48.4), 121.1 (43.0);<br>95.1 (100), 93.1 (46.6), 121.1 (45.9);<br>95.1 (100), 93.1 (54.5), 136.2 (43.2);<br>95.1 (100), 93.1 (46.5), 121.1 (45.6)	**1285**	1286<br>(0.1 ±<br>0.016)	1286<br>(0.3 ±<br>0.033)	1286<br>(0.1 ±<br>0.001)	1286<br>(0.6 ±<br>0.150)
**32**	2-Undecanone	26.9	112-12-9	$C_{11}H_{22}O$	*AK*	938 (949);<br>910 (922);<br>913 (923);<br>933 (941)	58.1 (100), 71.1 (37.9), 59.1 (30.0);<br>58.1 (100), 71.1 (38.2), 59.1 (29.8);<br>58.1 (100), 71.1 (39.3), 59.1 (30.0);<br>58.1 (100), 71.1 (37.8), 59.1 (29.8)	**1295**	1295<br>(0.7 ±<br>0.117)	1295<br>(0.2 ±<br>0.029)	1295<br>(0.5 ±<br>0.002)	1295<br>(0.6 ±<br>0.178)
33	2-Undecanol	27.2	1653-30-1	$C_{11}H_{24}O$	*OA*	922 (947);<br>892 (930);<br>902 (918);<br>874 (913)	45.1 (100), 55.1 (23.6), 83.1 (22.5);<br>45.1 (100), 55.1 (27.0), 83.1 (23.5);<br>45.1 (100), 83.1 (26.2), 83.1 (21.9);<br>45.1 (100), 55.1 (27.3), 83.1 (26.1)	**1301**	1302<br>(0.2 ±<br>0.032)	1302<br>(0.1 ±<br>0.011)	1302<br>(0.1 ±<br>0.001)	1302<br>(0.1 ±<br>0.020)
**34**	Elemene, δ-	28.7	20307-84-0	$C_{15}H_{24}$	*SH*	897 (900);<br>915 (915)	121.1 (100), 93.1 (65.9), 136.2 (61.5);<br>121.1 (100), 93.1 (64.7), 136.1 (53.7)	**1337**	NA	1337<br>(0.3 ±<br>0.248)	1337<br>(0.1 ±<br>0.001)	NA
**35**	Citronellyl acetate	29.5	150-84-5	$C_{12}H_{22}O_2$	*MAc*	806 (819);<br>903 (904);<br>905 (905);<br>901 (902)	95.1 (100), 81.1 (97.5), 69.1 (97.0);<br>81.1 (100), 95.1 (99.7), 69.1 (94.2);<br>95.1 (100), 81.1 (93.2), 69.1 (86.8);<br>81.1 (100), 95.1 (98.9), 69.1 (94.0)	**1355**	1355<br>(0.1 ±<br>0.038)	1355<br>(0.7 ±<br>0.217)	1355<br>(0.1 ±<br>0.001)	1355<br>(0.4 ±<br>0.090)
**36**	Cyclosativene	29.9	22469-52-9	$C_{15}H_{24}$	*SH*	897 (898);<br>884 (885);<br>851 (851);<br>918 (920)	105.1 (100), 161.2 (97.1), 204.3 (79.8);<br>105.1 (100), 161.2 (83.9), 119.1 (76.5);<br>161.2 (100), 105.1 (83.5), 119.1 (68.8);<br>105.1 (100), 161.2 (95.6), 119.1 (82.0)	**1362**	1363<br>(0.1 ±<br>0.024)	1363<br>(0.1 ±<br>0.038)	1363<br>(0.1 ±<br>0.009)	1363<br>(0.1 ±<br>0.023)
**37**	Copaene	30.4	3856-25-5	$C_{15}H_{24}$	*SH*	896 (896);<br>891 (891);<br>874 (874);<br>866 (866)	161.2 (100), 119.1 (95.3), 105.1 (86.3);<br>161.2 (100), 119.1 (95.7), 105.1 (85.6);<br>161.2 (100), 119.1 (95.3), 105.1 (86.3);<br>161.2 (100), 119.1 (93.4), 105.1 (83.2)	**1374**	1374<br>(0.2 ±<br>0.060)	1374<br>(0.3 ±<br>0.027)	1374<br>(0.3 ±<br>0.001)	1374<br>(0.2 ±<br>0.038)
**38**	Geranyl acetate	30.8	105-87-3	$C_{12}H_{20}O_2$	*MAc*	893 (905);<br>934 (937);<br>881 (899);<br>941 (942)	69.1 (100), 93.1 (46.9), 68.1 (41.6);<br>69.1 (100), 68.1 (38.8), 93.1 (34.3);<br>69.1 (100), 93.1 (52.4), 68.1 (37.7);<br>69.1 (100), 68.1 (38.6), 93.1 (34.4)	**1385**	1385<br>(0.2 ±<br>0.053)	1388<br>(6.4 ±<br>0.533)	1385<br>(0.2 ±<br>0.001)	1386<br>(2.3 ±<br>0.582)

Table S1 (continued)

**39**	Elemene, β-	31.1	515-13-9	$C_{15}H_{24}$	*SH*	883 (900); 877 (877); 877 (892); 885 (899)	93.1 (100), 81.1 (87.6), 67.1 (64.6); 93.1 (100), 81.1 (87.6), 67.1 (64.6); 93.1 (100), 81.1 (86.4), 107.1 (68.4); 93.1 (100), 81.1 (83.4), 107.1 (62.6)	**1391**	1391 (0.5 ± 0.152)	1391 (0.7 ± 0.053)	1391 (0.7 ± 0.018)	1391 (0.3 ± 0.079)
**40**	Sesquithujene	31.7	58319-06-5	$C_{15}H_{24}$	*SH*	897 (905); 905 (957); 899 (953); 911 (968)	93.1 (100), 119.1 (91.5), 91.1 (46.6); 119.1 (100), 93.1 (86.0), 91.1 (40.4); 119.1 (100), 93.1 (95.5), 91.1 (46.1); 119.1 (100), 93.1 (90.6), 91.1 (39.9)	**1405**	1406 (0.1 ± 0.014)	1406 (0.1 ± 0.009)	1406 (0.1 ± 0.001)	1406 (0.1 ± 0.027)
41	Caryophyllene	32.2	87-44-5	$C_{15}H_{24}$	*SH*	846 (846); 844 (844); 863 (863); 822 (824)	91.1 (100), 161.1 (94.4), 120.1 (76.6); 161.1 (100), 91.1 (90.0), 120.1 (78.4); 161.2 (100), 91.1 (76.4), 120.1 (76.0); 91.1 (100), 107.1 (72.9), 79.0 (61.3)	1417	1417 (0.1 ± 0.039)	1417 (0.1 ± 0.015)	1417 (0.1 ± 0.001)	1417 (0.1 ± 0.035)
**42**	Elemene, γ-	32.8	29873-99-2	$C_{15}H_{24}$	*SH*	919 (940); 923 (945); 917 (917); 846 (882)	121.1 (100), 93.1 (62.1), 107.1 (37.8); 121.1 (100), 93.1 (61.9), 107.1 (44.2); 121.1 (100), 93.1 (62.6), 107.1 (42.7); 121.1 (100), 93.1 (69.5), 107.1 (55.6)	**1433**	1433 (0.4 ± 0.090)	1433 (0.4 ± 0.041)	1433 (0.5 ± 0.009)	1433 (0.1 ± 0.011)
**43**	Germacrene D	34.7	23986-74-5	$C_{15}H_{24}$	*SH*	912 (933); 919 (936); 916 (934); 863 (891)	161.2 (100), 105.1 (54.1), 91.1 (47.0); 161.2 (100), 105.1 (54.5), 91.1 (46.5); 161.2 (100), 105.1 (54.9), 91.1 (48.1); 161.2 (100), 119.1 (86.4), 105.1 (72.7)	**1480**	1480 (0.9 ± 0.153)	1480 (1.2 ± 0.496)	1480 (1.5 ± 0.023)	1480 (0.3 ± 0.043)
**44**	Curcumene, α-	34.9	644-30-4	$C_{15}H_{22}$	*SH*	917 (936); 909 (937); 908 (932); 932 (940)	119.1 (100), 132.1 (87.6), 105.1 (52.5); 119.1 (100), 132.1 (86.7), 105.1 (53.1); 119.1 (100), 132.1 (87.5), 105.1 (53.9); 119.1 (100), 132.1 (88.8), 105.1 (50.3)	**1484**	1484 (2.6 ± 0.395)	1483 (2.2 ± 0.222)	1484 (3.6 ± 0.045)	1484 (4.5 ± 0.850)
**45**	Eudesmene, β-	35.2	17066-67-0	$C_{15}H_{24}$	*SH*	926 (950); 926 (950); 931 (931)	161.2 (100), 189.2 (68.8), 105.1 (67.4); 161.2 (100), 189.2 (68.6), 105.1 (67.6); 161.2 (100), 189.2 (72.2), 133.1 (68.8)	**1492**	1492 (0.6 ± 0.091)	1492 (0.7 ± 0.065)	1492 (0.9 ± 0.131)	NA
**46**	Zingiberene	35.5	495-60-3	$C_{15}H_{24}$	*SH*	861 (886); 902 (955); 896 (951); 898 (950)	119.1 (100), 93.1 (82.9), 91.1 (46.8); 119.1 (100), 93.1 (84.4), 91.1 (42.8); 119.1 (100), 93.1 (82.9), 91.1 (46.7); 119.1 (100), 93.1 (84.3), 91.1 (43.7)	**1499**	1499 (7.9 ± 1.132)	1498 (12.1 ± 1.003)	1497 (14.0 ± 1.288)	1499 (10.8 ± 1.863)

Table S1 (continued)

47	Bisabolene, β-	36.0	495-61-4	$C_{15}H_{24}$	*SH*	890 (899);<br>942 (948);<br>936 (942);<br>886 (886)	93.1 (100), 69.1 (56.3), 107.1 (48.1);<br>93.1 (100), 107.1 (52.6), 119.1 (47.2);<br>93.1 (100), 107.1 (54.9), 91.1 (46.5);<br>93.1 (100), 69.1 (66.1), 107.1 (44.0)	**1510**	1510<br>(4.6 ±<br>0.696)	1510<br>(7.4 ±<br>0.598)	1510<br>(8.4 ±<br>0.145)	1510<br>(4.6 ±<br>0.909)
**48**	Sesquiphellandrene<br>, β-	36.6	20307-83-9	$C_{15}H_{24}$	*SH*	906 (917);<br>936 (925);<br>899 (910);<br>912 (936)	69.1 (100), 93.1 (68.4), 91.1 (64.8);<br>69.1 (100), 93.1 (68.0), 91.1 (61.1);<br>69.1 (100), 93.1 (68.0), 91.1 (61.1);<br>69.1 (100), 93.1 (67.6), 91.1 (60.9)	**1525**	1525<br>(3.2 ±<br>0.511)	1526<br>(4.6 ±<br>0.468)	1526<br>(5.2 ±<br>0.065)	1526<br>(4.5 ±<br>0.967)
49	Bisabolene, γ-	36.8	53585-13-0	$C_{15}H_{24}$	*SH*	879 (930);<br>876 (912);<br>867 (900);<br>874 (907)	93.1 (100), 107.1 (99.5), 119.1 (48.2);<br>93.1 (100), 107.1 (94.6), 119.1 (49.4);<br>93.1 (100), 107.1 (90.6), 119.1 (52.0);<br>93.1 (100), 107.1 (94.6), 119.1 (44.8)	**1533**	1532<br>(0.1 ±<br>0.022)	1532<br>(0.2 ±<br>0.027)	1533<br>(0.2 ±<br>0.008)	1533<br>(0.1 ±<br>0.023)
**50**	Elemol	37.5	21657-90-9	$C_{15}H_{26}O$	*OS*	926 (929);<br>926 (929);<br>941 (946);<br>918 (921)	93.1 (100), 59.1 (98.7), 161.2 (80.1);<br>93.1 (100), 59.1 (96.9), 161.2 (81.9);<br>93.1 (100), 59.1 (91.3), 161.2 (85.4);<br>93.1 (100), 59.1 (97.7), 161.2 (80.8)	**1549**	1549<br>(0.9 ±<br>0.105)	1549<br>(1.2 ±<br>0.122)	1549<br>(0.7 ±<br>0.007)	1549<br>(0.3 ±<br>0.077)
**51**	Nerolidol	38.1	40716-66-3	$C_{15}H_{26}O$	*OS*	928 (932);<br>928 (932);<br>922 (925);<br>902 (909)	69.1 (100), 93.1 (80.7), 107.1 (47.3);<br>69.1 (100), 93.1 (81.5), 107.1 (47.7);<br>69.1 (100), 93.1 (86.3), 107.1 (49.4);<br>69.1 (100), 93.1 (81.9), 107.1 (47.2)	**1564**	1565<br>(0.8 ±<br>0.251)	1565<br>(0.8 ±<br>0.062)	1564<br>(0.7 ±<br>0.033)	1564<br>(0.2 ±<br>0.023)
**52**	Sesquisabinene<br>hydrate	39.0	58319-05-4	$C_{15}H_{26}O$	*OS*	891 (897);<br>890 (896);<br>879 (885);<br>886 (889)	69.1 (100), 93.1 (92.8), 119.1 (89.8);<br>69.1 (100), 93.1 (91.9), 119.1 (89.6);<br>93.1 (100), 69.1 (99.4), 119.1 (93.8);<br>69.1 (100), 93.1 (89.8), 119.1 (89.8)	**1590**	1589<br>(0.4 ±<br>0.061)	1589<br>(0.3 ±<br>0.048)	1589<br>(0.3 ±<br>0.022)	1590<br>(0.4 ±<br>0.092)
**53**	Eudesmol, γ-	40.1	1209-71-8	$C_{15}H_{26}O$	*OS*	880 (894);<br>881 (893);<br>858 (866);<br>899 (907)	161.2 (100), 189.2 (81.2), 81.1 (65.7);<br>161.2 (100), 189.2 (80.8), 81.1 (65.1);<br>161.2 (100), 189.2 (80.8), 81.1 (65.1);<br>189.2 (100), 161.2 (83.0), 133.1 (55.5)	**1620**	1618<br>(0.4 ±<br>0.074)	1618<br>(0.3 ±<br>0.008)	1618<br>(0.3 ±<br>0.035)	1618<br>(0.1 ±<br>0.032)

Table S1 (continued)

**54**	Muurolol, T-	41.0	19912-62-0	$C_{15}H_{26}O$	*OS*	820 (877);<br>822 (884);<br>803 (859)	161.2 (100), 95.1 (61.0), 105.1 (47.5);<br>161.2 (100), 95.1 (58.6), 105.1 (46.1);<br>161.2 (100), 95.1 (53.8), 204.2 (50.7)	**1641**	1641<br>(0.3 ±<br>0.040)	1641<br>(0.2 ±<br>0.034)	1641<br>(0.2 ±<br>0.029)	NA
**55**	Cubenol	41.1	21284-22-0	$C_{15}H_{26}O$	*OS*	810 (868);<br>834 (890);<br>813 (866)	161.2 (100), 119.1 (96.4), 95.1 (75.2);<br>161.2 (100), 119.1 (98.1), 95.1 (70.6);<br>161.1 (100), 119.1 (94.0), 105.0 (66.1)	**1646**	1646<br>(0.1 ±<br>0.040)	1646<br>(0.1 ±<br>0.019)	1646<br>(0.1 ±<br>0.006)	NA
**56**	Eudesmol, β-	41.2	473-15-4	$C_{15}H_{26}O$	*OS*	907 (918);<br>905 (914);<br>887 (895);<br>846 (869)	59.1 (100), 149.2 (85.2), 164.2 (43.1);<br>59.1 (100), 149.2 (86.6), 164.2 (44.8);<br>59.1 (100), 149.2 (99.1), 164.2 (50.4);<br>59.1 (100), 149.2 (86.1), 119.1 (54.0)	**1649**	1649<br>(0.8 ±<br>0.128)	1649<br>(0.6 ±<br>0.017)	1649<br>(0.6 ±<br>0.005)	1649<br>(0.4 ±<br>0.085)
**57**	Farnesol	43.9	106-28-5	$C_{15}H_{26}O$	*OS*	855 (889)	69.1 (100), 81.1 (33.2), 93.1 (29.8)	**1722**	NA	1722<br>(0.1 ±<br>0.023)	NA	NA
58	Farnesal	44.6	502-67-0	$C_{15}H_{24}O$	*SA*	905 (912);<br>903 (909);<br>892 (894);<br>890 (929)	69.1 (100), 84.1 (55.8), 81.1 (24.7);<br>69.1 (100), 84.1 (54.6), 81.1 (24.3);<br>69.1 (100), 84.1 (54.9), 81.1 (22.8);<br>69.1 (100), 84.1 (56.4), 81.1 (24.1)	**1740**	1742<br>(0.2 ±<br>0.049)	1742<br>(0.1 ±<br>0.031)	1742<br>(0.1 ±<br>0.007)	1742<br>(0.1 ±<br>0.027)
	**Total identified**								**82.6**	**83.8**	**84.6**	**87.4**

[a]Retention time of eluted compounds.
[b]Class of chemical compounds: *MH* monoterpenic hydrocarbon, *MO* monoterpenic oxide, *MA* monoterpenic aldehyde, *OM* monoterpenic alcohol, *MK* monoterpenic ketone, *MAc* monoterpenic acetate, *SH* sesquiterpenic hydrocarbon, *SA* sesquiterpenic aldehyde, *OS* sesquiterpenic alcohol, *AH* acyclic hydrocarbon, *AK* acyclic ketone, *AE* acyclic ester, *OA* acyclic alcohol.
[c]Matching scores of compounds reported ≥80% based on mass spectra in NIST library database and in the order of Bentong (BE), Cameron Highlands (CH), Sabah (SA) and Bara (BA) gingers.
[d]Fragmentation patterns reported in order of BE, CH, SA and BA.
[e]Retention index (RI) values calculated using Van Den Dool and Kratz equation with the reference to RI values from NIST WebBook and Adams (2007) within the range of ±10.
[f]Relative percentage abundance calculated on the basis of TIC (Total Ion Chromatogram) area as the percentage of total TIC area.
[g]NA: compound not available.

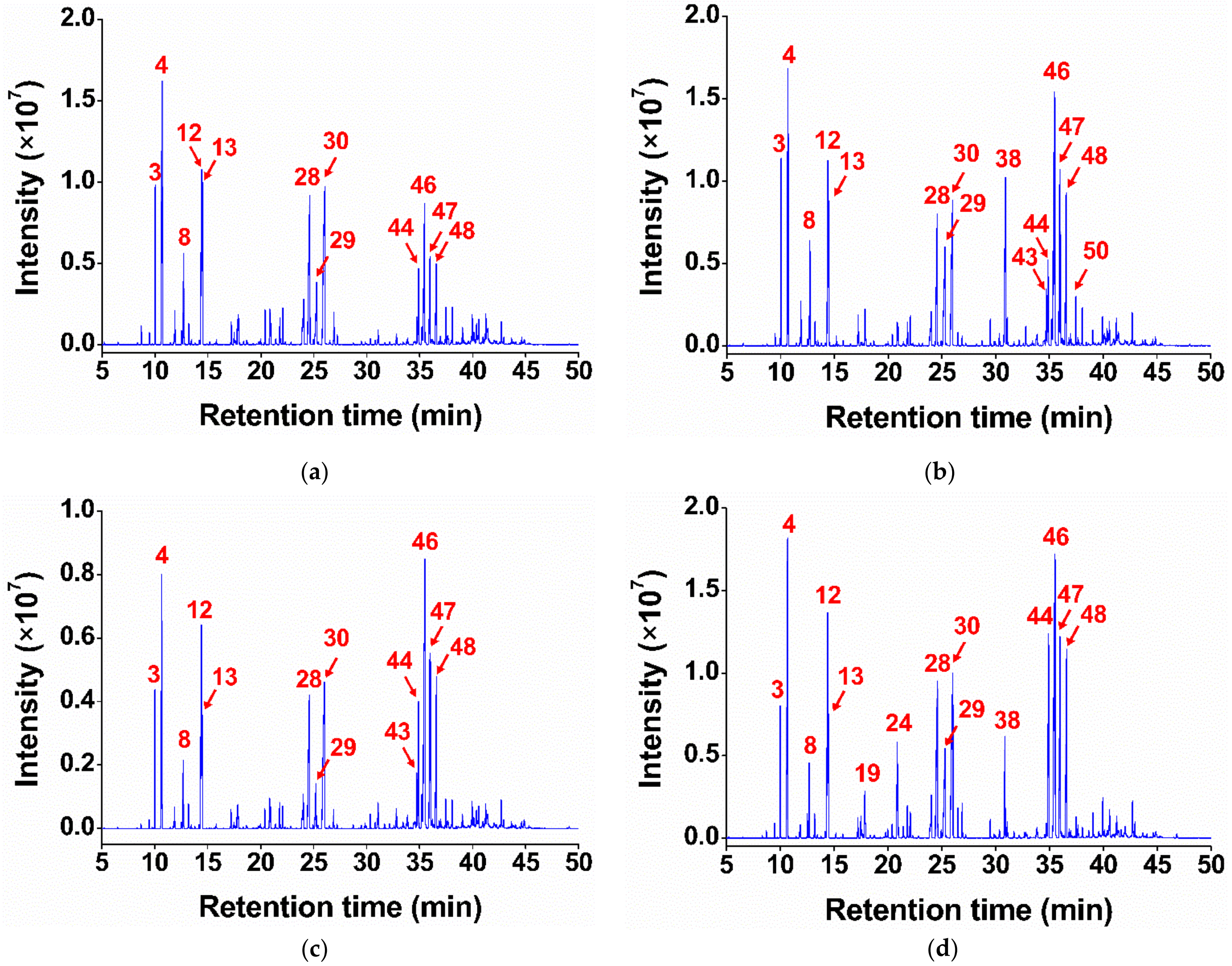

Figure S1. Total ion chromatograms (TICs) of the volatile profile of *Z. officinale* essential oil varieties. (a) Bentong, BE; (b) Cameron Highlands, CH; (c) Sabah, SA; and (d) Bara, BA. The peak numbering refers to Table S1.
